# Age-specific composition of milk microbiota in Tibetan sheep and goats

**DOI:** 10.1007/s00253-024-13252-w

**Published:** 2024-07-09

**Authors:** Xi Cao, Yumeng Fang, Pingcuo Bandan, Langda Suo, Gesang Jiacuo, Yujiang Wu, Awang Cuoji, Deqing Zhuoga, Yulin Chen, De Ji, Ciren Quzhen, Ke Zhang

**Affiliations:** 1 Key Laboratory of Animal Genetics, Breeding and Reproduction of Shaanxi Province, College of Animal Science and Technology, Northwest A&F University, Xianyang, 712100 Yangling China; 2 Institute of Animal Sciences, Tibet Academy of Agricultural and Animal Husbandry Sciences, Lhasa, 850009 China

**Keywords:** Milk microbiota, Ruminant, Colostrum, Milk composition, *Lactobacillus*

## Abstract

**Abstract:**

This study investigates the dynamic changes in milk nutritional composition and microbial communities in Tibetan sheep and goats during the first 56 days of lactation. Milk samples were systematically collected at five time points (D0, D7, D14, D28, D56) post-delivery. In Tibetan sheep, milk fat, protein, and casein contents were highest on D0, gradually decreased, and stabilized after D14, while lactose and galactose levels showed the opposite trend. Goat milk exhibited similar initial peaks, with significant changes particularly between D0, D7, D14, and D56. 16S rRNA gene sequencing revealed increasing microbial diversity in both species over the lactation period. Principal coordinates analysis identified distinct microbial clusters corresponding to early (D0–D7), transitional (D14–D28), and mature (D56) stages. Core phyla, including *Proteobacteria*, *Firmicutes*, *Bacteroidetes*, and *Actinobacteria*, dominated the milk microbiota, with significant temporal shifts. Core microbes like *Lactobacillus*, *Leuconostoc*, and *Streptococcus* were common in both species, with species-specific taxa observed (e.g., *Pediococcus* in sheep, *Shewanella* in goats). Furthermore, we observed a highly shared core microbiota in sheep and goat milk, including *Lactobacillus*, *Leuconostoc*, and *Streptococcus*. Spearman correlation analysis highlighted significant relationships between specific microbial genera and milk nutrients. For instance, *Lactobacillus* positively correlated with total solids, non-fat milk solids, protein, and casein, while *Mannheimia* negatively correlated with protein content. This study underscores the complex interplay between milk composition and microbial dynamics in Tibetan sheep and goats, informing strategies for livestock management and nutritional enhancement.

**Key points:**

*• The milk can be classified into three types based on the microbiota composition*

*• The changes of milk microbiota are closely related to the variations in nutrition*

*• Filter out microbiota with species specificity and age specificity in the milk*

**Supplementary Information:**

The online version contains supplementary material available at 10.1007/s00253-024-13252-w.

## Introduction

Breast milk is the exclusive nutritional source for pre-weaning lambs, providing essential proteins, carbohydrates, fats, urea, nucleotides, and other non-protein nitrogenous components. These nutrients dynamically change throughout lactation to meet the specific needs of lambs, promoting their early growth, development, and immune health (Chen et al. 2018b, c, a; Yadav et al. 2022). The complex composition of breast milk highlights its role as a biologically intricate fluid, closely tied to the physiology of both the mother and the lamb (Andreas et al. 2015). Disruptions in this system can significantly affect lamb growth, development, and maternal well-being (Brodin 2022). One often overlooked aspect of breast milk is its substantial microbial content. Daily, offspring ingest milk containing about 8 × 105 bacteria, which may directly contribute to the gastrointestinal microbiota in young animals, providing foundational “seeds” for gut microbiota development (Le Doare et al. 2018; Moubareck 2021). This microbial component is crucial for shaping developmental trajectories, with disruptions potentially leading to long-lasting effects. Studies suggest that breast milk contributes approximately 25 to 30% of the microbial source during the establishment of early gut microbiota in offspring (Pannaraj et al. 2017). The interaction of milk microbiota and lactose can mitigate developmental delays, enhance weight gain, and reduce the risk of gastrointestinal diseases in young animals (Charbonneau et al. 2016).

The lactation period impacts both the nutrient composition and the microbial content of milk (Qin et al. 2021). The microbial composition of milk varies across different lactation stages, from colostrum to mature milk, affecting the developing gut microbiota of offspring (Fitzstevens et al. 2017). Colostrum, rich in immunoglobulins and beneficial microbes, is crucial for the initial establishment of the neonatal gut microbiota, while mature milk supports the continued development and stabilization of these microbial communities (Kalbermatter et al. 2021). In ruminant animals like goats and sheep, the rumen undergoes distinct stages of microbial colonization, influencing the digestion and utilization efficiency of post-weaning diets (Zhang et al. 2019). Understanding milk microbiota succession dynamics is crucial for shaping gut microbiota colonization in lambs, as it supports the development of the rumen microbiota, which is essential for digesting complex plant materials post-weaning.

Host genetics also significantly influence milk microbiota composition. Comparative analysis shows that roe deer milk has the highest bacterial diversity, followed by reindeer milk, with goat milk having the lowest (Li et al. 2017). Roe deer milk is rich in *Pseudomonas* and *Acinetobacter* bacteria, while reindeer and goat milk are dominated by unclassified bacteria from the *Microbacteriaceae* family and *Bacillus* genus, respectively. The presence of the *Salinicoccus* genus in all three types of milk reveals both common and unique characteristics of bacterial communities in the milk niche. This uniqueness in roe deer and reindeer milk may reflect evolutionary adaptations of host microbiota (Li et al. 2017).

Despite promising insights, previous research on milk microbiota changes with lactation age has been limited by extended sampling intervals, necessitating further investigation. While microbial succession in human milk and its implications for infant health have been documented (Huertas-Díaz et al. 2023; Stinson and Geddes 2022), research on livestock, particularly Tibetan goats and sheep, remains sparse. Our study addresses this gap by focusing on Tibetan goats and sheep, employing frequent sampling across colostrum, transitional milk, and mature milk. Our aim is to elucidate the patterns of milk microbiota changes with lactation age and identify milk microbiota that can modulate the early rumen microbial community. By utilizing functional milk replacers and feed alternatives, we hope to enhance intestinal development and microbial colonization, ultimately improving lamb health and productivity. This study seeks to explore the dynamics of milk microbiota succession in Tibetan goats and sheep, its impact on gut microbiota colonization in lambs, and the potential of milk replacers and feed alternatives to boost lamb health and productivity.

## Materials and methods

### Ethical approval

The experiment was conducted following the guidelines and regulations set forth by the Institutional Animal Care and Use Committee of the Northwest A&F University, and it was approved under permit number 202105509.

### Experimental animal selection and management

Pengbo sheep and Tibetan goats, devoid of any history of antibiotic treatment, were selected as experimental subjects. Throughout the experiment, all animals remained healthy, and no signs of mastitis were observed. Detailed information about the animals, including birth date, lambing date, lambing number, age at lambing (year), sampling date, and days in milk, is provided in Table S1. The diets of sheep and goats were managed at the same level and remained unchanged during the trial period. The diet composition and nutritional levels of the animals are presented in Table S2.

### Sample collection

Milk samples were collected regularly from the day of lambing (designated as D0) until the 56th day of lambing. Specifically, milk samples were collected from maternal goats and sheep on days D0, D7, D14, D28, and D56. Each animal’s mammary glands were carefully milked into a sterile container, and the collected milk was divided into two 50-mL sterile tubes. The udder and nipples were thoroughly cleaned with sterile wet wipes and 75% ethanol, respectively, before sample collection. The initial milk drops from each goat and sheep were discarded to ensure proper collection. The samples were labeled immediately after collection, and one aliquot was transported to the lab in an ice box for milk composition analysis, while the other was promptly stored in liquid nitrogen for subsequent metagenomic DNA extraction. During the experimental period, we collected a total of 63 Tibetan sheep milk samples and 75 Tibetan goat milk samples. The determination of milk sampling times was based on our prior research into the colonization patterns of gastrointestinal microbiota in sheep and goats (Guo et al. 2020). We previously identified D0, D7, D14, D28, and D56 as critical stages for rumen microbial colonization (Li et al. 2019; Wang et al. 2019; Zhang et al. 2019). Furthermore, in current intensive farming practices of sheep and goats, lambs are typically weaned after 56 days; hence, we did not extend our study beyond this period to investigate milk microbiota. Throughout the experiment, both sheep and goats exhibited healthy udders without any signs of clinical mastitis, and somatic cell counts in milk samples ranged from 34,400 to 417,000 cells/mL.

### Analysis of milk nutrient composition

The milk samples were gently mixed before analysis to ensure homogeneity. Milk fat, protein, non-fat milk solids (SNF), total solids, lactose, low lactose, galactose, and casein content were quantified using the YWAY-CP2 automatic milk composition analyzer.

### Metagenomic DNA extraction of milk bacteria

To achieve accurate microbial diversity characterization, metagenomic DNA was extracted from each 50 mL milk sample. The milk samples were centrifuged at 10,000 r/min (4 °C) for 10 min, as they had relatively low microbial levels. Genomic DNA from the milk’s microbes was extracted using the E.Z.N.A. soil DNA kit (Omega Bio-tek, Norcross, GA, USA), and the quality and concentration of the DNA were assessed using NanoDrop2000 (Thermo Scientific, USA). The 16S rRNA gene V3-V4 variable region was amplified by PCR using primers 338F (5′-ACTCCTACGGGAGGCAGCAG-3′) and 806R (5′-GGACTACHVGGGTWTCTAAT-3′) containing barcode sequences. Sequencing of the purified amplicons was performed on an Illumina MiSeq platform (Illumina, San Diego, USA) using paired-end sequencing (2 × 300 bps) with equimolar ratios of the pooled amplicons.

### Statistical analysis

GraphPad Prism (V 9. GraphPad, USA) was used to draw column line charts, and statistical data were presented as the mean ± standard error of the mean (SEM). One-way analysis of variance (one-way ANOVA) and Tukey’s multiple comparisons test (IBM SPSS Statistics 27.0) were applied to analyze alpha diversity and milk composition data. The Shannon index was utilized to calculate alpha diversity at the ASV level, and the Wilcoxon rank-sum test was used to analyze significant differences in alpha diversity indices among different days. Beta diversity indices (Bray–Curtis) were calculated in QIIME2 (R-3.3.1, vegan) using Bray–Curtis distance similarity analysis (ANOSIM) to assess statistical significance in microbial community variations across milk samples collected at different time points. Differences in the relative abundance of milk microbes at various time points were analyzed using the Kruskal–Wallis *H* test on the Majorbio cloud platform (www.majorbio.com). A heat map analysis using Spearman correlation (*P* < 0.05) was performed to assess the association between milk nutrient content and bacteria. Software BugBase was used to predict the behavior of milk microbial phenotypes (*P* < 0.05). *P*-values for milk microbiota data were transformed into false discovery rates (FDR) using the Benjamini–Hochberg method.

## Result

### Changes in milk nutrition composition from D0 to D56 days in Tibetan sheep and goat

In this study, milk samples were systematically collected at different time points (days 0, 7, 14, 28, and 56) post-delivery from both sheep and goat ewes. Traditional nutrient fractions present in milk were meticulously analyzed, revealing significant variations. Dynamic analysis of the nutritional components in sheep milk from day 0 to day 56 of lactation revealed that the contents of milk fat, milk protein, and casein were highest on D0, gradually decreased, and then remained relatively stable after D14. In contrast, the contents of lactose, galactose, and low lactose showed the opposite trend (Table 1). Dynamic analysis of the nutritional components in goat milk from day 0 to day 56 of lactation revealed that on D0, the contents of milk fat, protein, non-fat milk solids, total milk solids, low lactose, galactose, and casein were the highest. Milk fat content gradually decreased with the increase in lactation days and can be divided into three stages: D0, D7–D14, and D28–D56, with significant differences in milk fat content among these stages (*P* < 0.001). The contents of protein, casein, and non-fat milk solids showed significant differences between D0 and D7 (*P* < 0.001). The content of galactose did not differ significantly across the five time points. After D7, the content of low lactose did not show significant changes (*P* > 0.05); galactose content significantly decreased after D28. Compared to D0 and D7, casein content began to significantly decrease from D14, and there were no significant differences after D14 (*P* > 0.05; Table 2).

**Table 1:** Changes in nutrient composition of milk during the lactation period of sheep

Group	Fat (%)	Protein (%)	SNF (%)	Solids (%)	Lactose (%)	Low lactose (%)	Galactose (%)	Casein (%)
D0	10.39 ± 2.35^a^	8.03 ± 2.09^a^	13.44 ± 2.08^a^	23.36 ± 2.54^a^	4.02 ± 0.52^b^	2.98 ± 0.81^c^	0.33 ± 0.10^b^	5.53 ± 1.43^a^
D7	8.67 ± 1.74^b^	7.51 ± 0.74^a^	13.49 ± 0.83^a^	20.82 ± 1.52^b^	4.33 ± 0.34^b^	4.15 ± 0.50^b^	0.35 ± 0.10^b^	5.51 ± 0.51^a^
D14	6.89 ± 1.54^c^	6.22 ± 0.92^b^	12.06 ± 0.61^b^	18.52 ± 1.04^c^	4.89 ± 0.59^a^	4.95 ± 0.43^a^	0.28 ± 0.12^b^	4.58 ± 0.63^b^
D28	7.49 ± 1.29^bc^	5.86 ± 0.79^b^	11.91 ± 0.81^b^	18.82 ± 1.88^c^	5.08 ± 0.22^a^	4.85 ± 0.25^a^	0.54 ± 0.16^a^	4.61 ± 0.64^b^
D56	8.09 ± 1.70^bc^	6.43 ± 0.63^b^	12.59 ± 1.09^ab^	20.65 ± 1.96^b^	5.05 ± 0.29^a^	4.90 ± 0.61^a^	0.57 ± 0.22^a^	4.08 ± 0.53^b^
SEM	0.29	0.20	0.19	0.35	0.08	0.13	0.03	0.13
*P* value	< 0.001	< 0.001	0.009	< 0.001	< 0.001	< 0.001	< 0.001	0.007

The data in the table represents the percentage of various nutritional components in the milk. Data are presented as the mean ± SEM. Different letters in the same column indicate significant differences between the two groups (*P* < 0.05), and the same letters indicate that the differences between the two groups are not significant (*P* > 0.05)

**Table 2:** Changes in nutrient composition of milk during the lactation period of Tibetan goats

Group	Fat (%)	Protein (%)	SNF (%)	Solids (%)	Lactose (%)	Low lactose (%)	Galactose (%)	Casein (%)
D0	8.93 ± 0.28^a^	11.99 ± 0.26^a^	17.36 ± 0.35^a^	24.80 ± 0.52^a^	4.40 ± 0.09^ab^	3.30 ± 0.18^a^	1.32 ± 0.08^a^	8.68 ± 0.28^a^
D7	7.50 ± 0.09^b^	9.52 ± 0.09^b^	14.95 ± 0.19^b^	19.64 ± 0.66^b^	3.99 ± 0.08^b^	2.58 ± 0.10^b^	1.29 ± 0.10^a^	6.36 ± 0.37^b^
D14	6.70 ± 0.13^b^	5.91 ± 0.18^c^	12.14 ± 0.27^c^	18.16 ± 0.94^bc^	4.16 ± 0.34^ab^	2.46 ± 0.17^b^	1.01 ± 0.18^a^	4.31 ± 0.25^c^
D28	5.28 ± 0.09^c^	5.37 ± 0.15^c^	12.18 ± 0.12^c^	17.39 ± 0.38^c^	4.14 ± 0.27^ab^	2.15 ± 0.28^b^	0.26 ± 0.16^b^	3.56 ± 0.17^c^
D56	5.70 ± 0.09^c^	5.21 ± 0.15^c^	11.16 ± 0.24^c^	17.00 ± 0.24^c^	4.86 ± 0.17^a^	2.46 ± 0.07^b^	0.29 ± 0.05^b^	3.93 ± 0.12^c^
SEM	0.22	0.44	0.40	0.52	0.10	0.08	0.08	0.32
*P* value	< 0.001	< 0.001	< 0.001	< 0.001	0.007	< 0.001	< 0.001	< 0.001

The data in the table represents the percentage of various nutritional components in the milk. Data are presented as the mean ± SEM. Different letters in the same column indicate significant differences between the two groups (*P* < 0.05), and the same letters indicate that the differences between the two groups are not significant (*P* > 0.05)

**Fig. 1:**
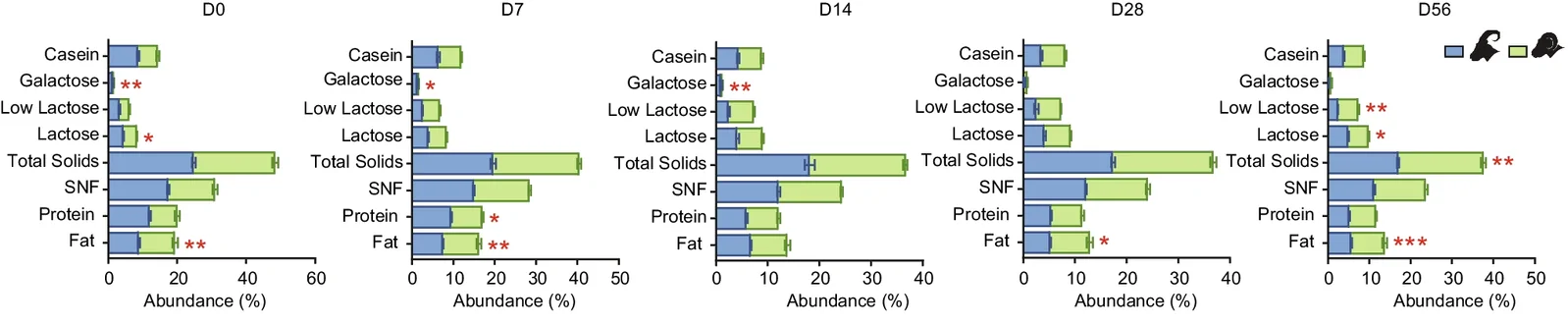
Comparison analysis of milk nutrient components at the same lactation days in Tibetan goats and sheep. Comparison analysis of milk fat, protein, non-fat milk solids (SNF), total solids, lactose, low lactose, galactose, and casein in Tibetan Sheep and Goats during D0–D0. Statistical assessment of data differences in the nutritional components of sheep and goat milk using one-way analysis of variance (ANOVA). Goats are denoted in blue, while sheep are represented in green. Statistical significance levels are denoted as follows: * for *P* < 0.05, ** for *P* < 0.01, and *** for *P* < 0.001

Based on these observed variations, the milk from sheep and goats was categorized into two distinct lactation subtypes: colostrum type (days 0–7) and standing milk type (days 14–56). These alterations in nutrient fractions underscore substantial shifts in the nutritional composition of sheep and goat milk over the 0–56-day period. Additionally, a comparative analysis of the nutritional composition of sheep and goat milk under similar lactation day conditions revealed specific differences. At various time points, significant disparities were noted. Specifically, sheep milk exhibited significantly higher fat content than goat milk on days 0, 7, 28, and 56 (*P* < 0.05; Fig. 1). Furthermore, sheep milk displayed significantly higher total solids content compared to goat milk on D56 (*P* < 0.05; Fig. 1), although no significant differences were observed on other lactation days. Lactose levels in sheep milk were significantly lower than in goat milk on D0 (*P* < 0.05) and significantly higher on D56 (*P* < 0.05; Fig. 1), with no significant differences on other lactation days. Additionally, galactose content in sheep milk was significantly lower than in goat milk on days 0, 7, and 14 (*P* < 0.05), whereas no significant differences were observed on days 28 and 56. Notably, protein levels in sheep milk were significantly lower than in goat milk on D7 (*P* < 0.05; Fig. 1), with no significant differences noted on other lactation days. These findings illuminate the dynamic changes in milk composition during the lactation period in Tibetan sheep and goats.

### Succession process of lactation milk microbial composition in goats and sheep

**Fig. 2:**
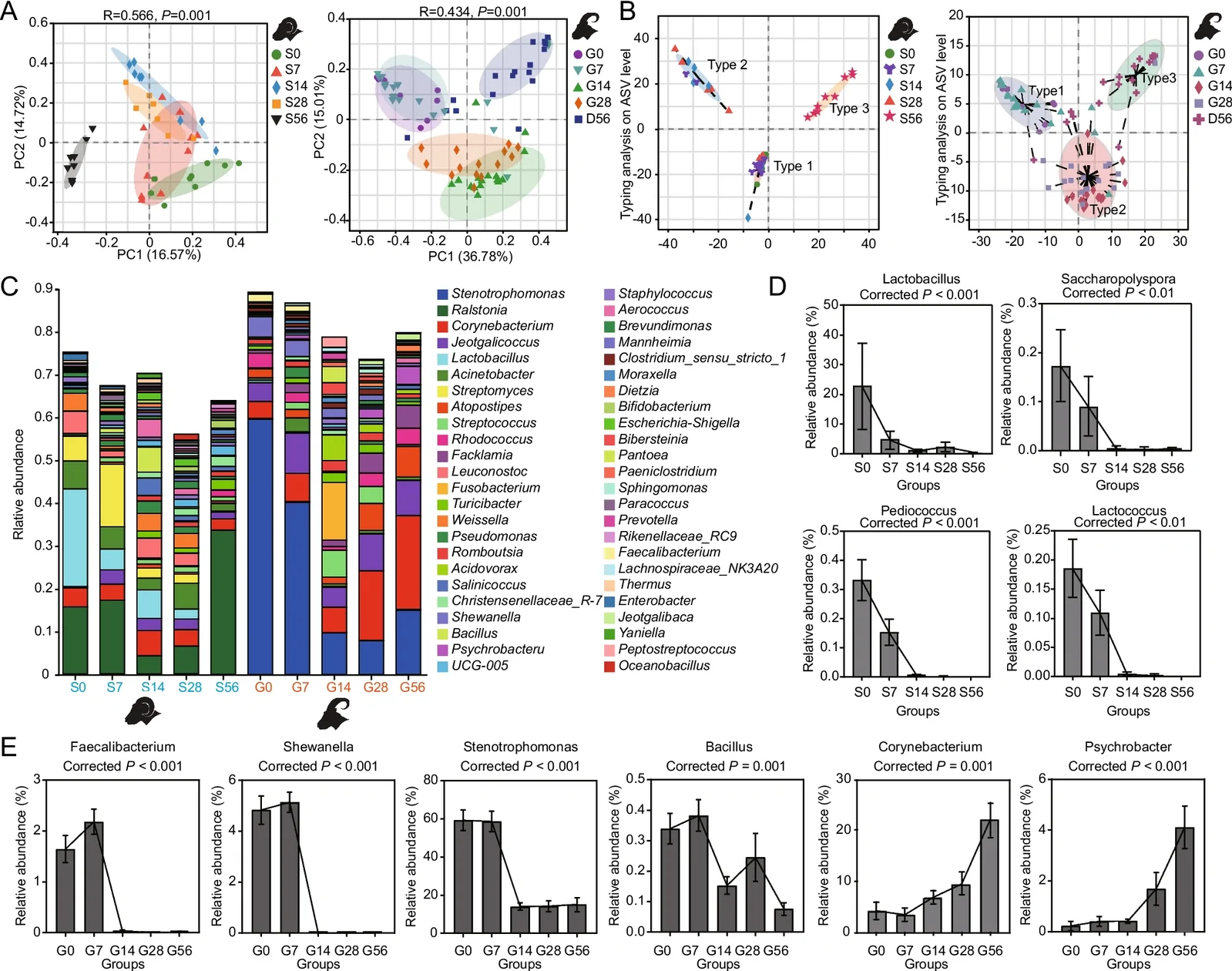
Succession process of lactation milk microbial composition in Tibetan goats and sheep. **A** The principal coordinate analysis (PCoA) plot on the ASV matrix showed the β-diversity of the sheep and goat’s milk microbiota. β-diversity was determined using ANOSIM analysis. **B** Typing analysis of microbial communities in the milk of sheep and goats at the ASV Level. **C** At the genus level, the composition and relative abundance of microbial communities in the milk of sheep and goats were assessed. **D** Microorganisms specifically present in sheep colostrum. **E** Microorganisms specifically present in Type 1 goat milk and the relative abundance changes of key bacterial taxa

We sequenced the 16S rRNA gene in both sheep and goat milk to investigate how the bacterial communities in the milk changed over the course of the lactation period. Our findings revealed an increasing trend in the Shannon index for milk bacteria in sheep and goats from D0 to D56, as depicted in Fig. S1A. Utilizing the Bray–Curtis distance algorithm and Principal Coordinates Analysis (PCoA) at the Amplicon sequence variant (ASV) level, we identified three distinct clusters in the microbial composition at five time points in both sheep and goats. Specifically, D0 and D7 exhibited significant clustering of milk microbes, as did D14 and D28, while D56 exhibited significant separation from the other time periods (*R* = 0.566, *P* = 0.001; *R* = 0.434, *P* = 0.001, respectively; Fig. 2A). This clustering pattern suggests shifts in the milk microbiota composition over time. Furthermore, through the application of clustering software, our analysis revealed that the lactation period of sheep and goats, spanning from 0 to 56 days, can be categorized into three types. Type 1 primarily corresponds to the early lactation stage (D0–D7), Type 2 represents a transitional phase (D14–D28), and Type 3 characterizes the mature stage (D56), as illustrated in Fig. 2B.

Upon taxonomic assignment, we annotated the milk microbial sequences from sheep and goats to 50 phyla (Table S3), 629 families (Table S4), and 1663 genera (Table S5). Our analyses at various taxonomic levels unveiled four core phyla (with relative abundance ≥ 1%) in both sheep and goats, namely *Proteobacteria*, *Firmicutes*, *Bacteroidetes*, and *Actinobacteria*. These four phyla collectively accounted for over 90% of the total bacterial abundance, while phyla such as *Chloroflexi*, *Acidobacteriota*, and *Verrucomicrobiota* were considered non-core, with a relative abundance of less than 1% (Fig. S1B). To gain a deeper understanding of the shared microbial composition changes in sheep and goat milk during the 0–56-day period, we conducted multiple group comparisons using the Wilcoxon rank-sum test. At the phylum level, we observed specific trends, such as a decrease in the relative abundance of *Acidobacteriota* during the D0–D14 stage, followed by an increase, and then another decrease. Additionally, the relative abundance of *Proteobacteria* significantly decreased at D14 (*P* < 0.01), while *Verrucomicrobiota* exhibited a significant decrease from D28 to D56 (*P* < 0.01), and *Patescibacteria* showed a significant decrease from D28 to D56 (*P* < 0.05; Fig. S2).

At the genus level, our analysis identified 875 shared genera in both sheep and goat milk, with *Stenotrophomonas*, *Ralstonia*, *Corynebacterium*, *Jeotgalicoccus*, *Lactobacillus*, *Streptomyces*, *Rhodococcus*, *Leuconostoc*, *Acinetobacter*, *Facklamia*, and *Shewanella* exhibiting a relative abundance ≥ 1% (Fig. 2C). Among these, *Jeotgalicoccus* and *Leuconostoc* maintained stable relative abundances across all five time points, while other shared genera displayed day-age and species variability (Fig. 2C). Upon further examination of microbial differences in milk profiles, noteworthy distinctions emerge. In sheep milk, the genera *Lactobacillus*, *Lactococcus*, *Pediococcus*, and *Saccharopolyspora* were exclusively detected in Type 1 milk, while they were absent in Type 2 and Type 3 milk (Fig. 2D). In goat milk, the specific genera present in Type 1 milk included *Shewanella* and *Faecalibacterium* (Fig. 2E). In goat milk, the abundance of *Corynebacterium*, *Psychrobacter*, *Facklamia*, and *Prevotellaceae_UCG-001* gradually increases with the days of lactation, and *Stenotrophomonas* exhibits a high abundance in Type 1 milk, while in Type 2 and Type 3 milk, its abundance decreases and remains stable (Fig. 2E, S3). In sheep milk, the abundance of *Akkermansia*, *Prevotellaceae_UCG-004*, *Christensenellaceae_R-7_group*, and *Bifidobacterium* gradually increases with the days of lactation (Fig. S3). This further confirms the species specificity of microbial composition in sheep and goat milk.

### Prediction of particular lactation characteristics between Tibetan sheep and goats

**Fig. 3:**
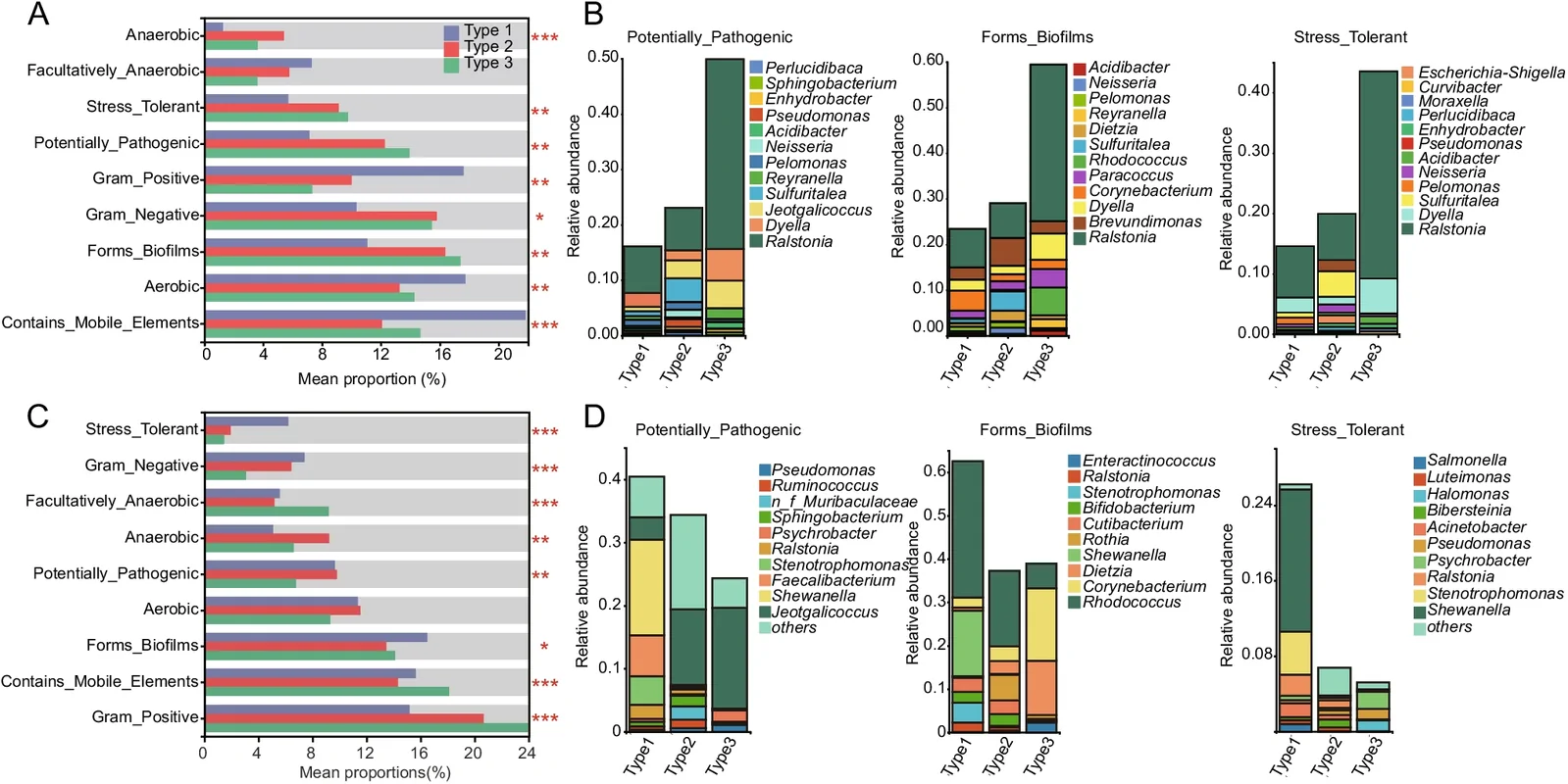
Phenotypic functional prediction of microbial communities in different milk types of Tibetan sheep and goats. Utilizing BugBase to predict the phenotypes of dominant microorganisms in different milk types of sheep (**A**) and goats (**C**), including gram-positive, gram-negative, biofilm formation, pathogenic, mobile element containing, oxygen utilizing, and oxidative stress tolerant. Statistical significance levels are denoted as follows: * for *P* < 0.05, ** for *P* < 0.01, and *** for *P* < 0.001. Species-phenotype contribution analysis of specific phenotypes in the milk microbial communities of sheep (**B**) and goats (**D**), including, potential pathogenicity, biofilm formation, and stress tolerance

The variation in the core microbial composition and abundance in milk is intricately associated with their functional roles. To elucidate the phenotypic functions of the microbial communities in the three milk types, we employed BugBase for microbial phenotype prediction. Notably, the relative abundance of anaerobic bacteria was found to be significantly higher in Type 2 milk of both sheep and goats compared to Type 1 and Type 3 milk (*P* < 0.05; Fig. 3A, C). In order to explore the relationship between milk microbiota and phenotypes, specific phenotypes including stress tolerant, potentially pathogenic, and biofilm formation were selected for species-phenotype contribution analysis. The findings revealed that in Type 1 milk of both sheep and goats, the primary contributing bacterial genus for stress tolerant and potentially pathogenic phenotypes was *Ralstonia*, while the main contributing bacterial genera for the biofilm formation phenotype were *Rhodococcus*, *Dietzia*, *Corynebacterium*, *Brevundimonas*, and *Ralstonia* (Fig. 3B, D). In Type 2 milk, the major contributing bacterial genera for stress tolerant were *Pseudomonas* and *Ralstonia*; for potentially pathogenic were *Moraxella*, *Enhydrobacter*, *Pseudomonas*, and *Ralstonia*; and for biofilm formation were *Bifidobacterium*, *Brachybacterium*, *Corynebacterium*, *Pseudomonas*, *Brevundimonas*, and *Ralstonia* (Fig. 3B, D). Meanwhile, in Type 3 milk, the key contributing bacterial genera for stress tolerant were *Pseudomonas* and *Ralstonia*; for potentially pathogenic were *Enhydrobacter* and *Ralstonia*; and for biofilm formation were *Dietzia*, *Corynebacterium*, and *Ralstonia* (Fig. 3B, D). These results shed light on the functional attributes of microbial communities in different milk types, emphasizing their potential roles in stress tolerance, pathogenicity, and biofilm formation. The observed variations underscore the dynamic and phenotype-specific nature of the interactions between milk microbiota and their functional roles in sheep and goat milk.

### The relationship between the microbiota and nutrient content of Tibetan sheep and goat milk

**Fig. 4:**
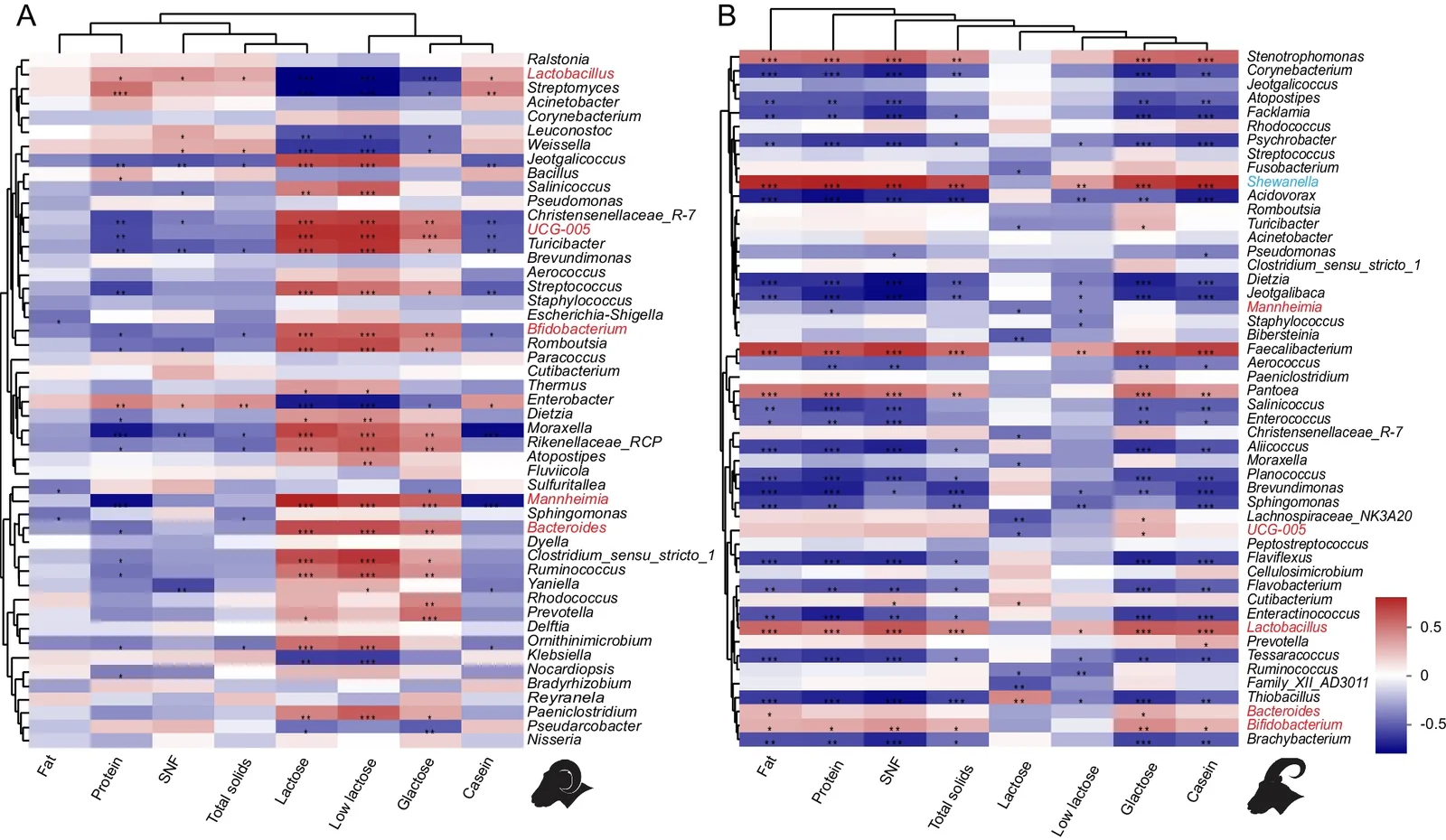
The relationship between the microbiota and nutrient content of Tibetan sheep and goat milk. Exploring the potential relationship between the relative abundance changes of milk microbiota and variation in milk components in sheep (**A**) and goats (**B**) through Spearman correlation analysis

To explore potential relationships between changes in milk microbiota and variations in milk nutrient components, Spearman correlation analysis was employed to examine the association between milk microbiota and milk nutrient composition. The results revealed significant positive correlations (*r* > 0.4, *P* < 0.05) between the relative abundance of *Lactobacillus* in both sheep and goat milk and the content of total solids, SNF, protein, and casein. Conversely, a significant negative correlation (*r* <  − 0.4, *P* < 0.05) was observed between the relative abundance of *Mannheimia* and the protein content. *Salinicoccus* exhibited a significant negative correlation (*r* <  − 0.4, *P* < 0.05) with the SNF content. Furthermore, the relative abundances of *Bacteroides* and *Bifidobacterium* showed a significant positive correlation (*r* > 0.4, *P* < 0.05) with low lactose content. *UCG-005*, *Bacteroides*, and *Bifidobacterium* displayed a significant positive correlation (*r* <  − 0.4, *P* < 0.05) with galactose content (Fig. 4). In summary, the variations in the relative abundance of core microbiota in both sheep and goat milk appear to be influenced by changes in the content of conventional nutrient components in the milk. These correlations suggest potential interdependencies between specific microbial genera and key nutrient components, providing valuable insights into the intricate relationship between milk microbiota and its nutritional composition.

## Discussion

In this study, we collected milk samples from sheep and goats at 0, 7, 14, 28, and 56 days after parturition and analyzed the routine nutritional components of the milk as well as performed microbial 16S rRNA gene sequencing. We observed the microbial profiles of sheep and goat milk exhibited distinct clustering patterns at the five time points, forming three distinct clusters: Type 1 (D0–D7), Type 2 (D14–D28), and Type 3 (D56), and identified 34 bacterial phyla and 875 bacterial genera shared between sheep and goat milk. The composition of milk microbiota in both sheep and goat milk underwent significant changes from D0 to D56. Additionally, there were specific bacterial taxa that were either specific to certain species or exhibited temporal specificity in sheep and goat milk.

Colostrum plays a pivotal role in the growth, development, and immune health of young mammals, with its nutritional composition exhibiting temporal specificity. Previous studies have consistently reported a gradual decrease in the percentages of protein, fat, total solids, and lactose from colostrum to mature milk (Akinsoyinu et al. 1977; Timlin et al. 2021). In our investigation, we observed significantly higher levels of fat, protein, SNF, and total solids in colostrum compared to transition milk and mature milk in both sheep and goats. As the transition from colostrum to mature milk progressed, there was a gradual decline in the levels of fat, protein, and casein essential macronutrients. Conversely, the lactose content exhibited an increasing trend during this transition. This observed pattern aligns with findings from previous studies conducted on various species, including Laoshan dairy sheep (Chen et al. 2018a), Assaf sheep (Toral et al. 2010), horses (Barreto Í et al. 2020), cows (Lim et al. 2020), and humans (Neville et al. 1991). Moreover, our study revealed that the fat content in sheep milk surpassed that in goat milk during both colostrum and mature milk stages. However, in colostrum, the content of protein, SNF, total solids, lactose, and casein in Tibetan sheep milk was slightly lower than that in goats (Pastuszka et al. 2016). These variations in milk composition, especially during the transition from colostrum to mature milk, underline the dynamic nature of nutritional components and highlight the uniqueness of the Tibetan sheep milk profile. The observed differences may be attributed to the specific environmental conditions and geographical factors inherent to the Tibetan region.

The specificity of colostrum contributes to its unique microbial composition, as identified in our research on Tibetan sheep and goats. Notably, *Lactococcus*, a common beneficial microorganism found in milk, exhibits immune-regulatory activities, facilitates the colonization of beneficial gut bacteria, and enhances animal productivity (Yu et al. 2021). Research indicates that *Lactococcus* has immunomodulatory and potential therapeutic effects, promoting tissue recovery after airway inflammation in mice (Yang et al. 2015). Additionally, its fermentation fluid enhances the antioxidant function of serum and liver in mice (Lee et al. 2022). Most species in the genus *Pediococcus*, identified in our study, act as probiotics with inhibitory effects against foodborne pathogens and antifungal properties (Kamiloğlu 2022). They contribute to regulating intestinal immune function, lowering cholesterol levels, and resisting tumors (Han et al. 2021). *Saccharopolyspora*, a gram-positive bacterium, produces various active substances such as antibiotics, vitamins, enzymes, and algal growth factors (Veyisoglu et al. 2022). Specific strains like *Saccharopolyspora hirsuta* and *Saccharopolyspora erythraea* produce antibiotics effective in inhibiting the proliferation of pathogenic bacteria in milk (Thompson et al. 2002). *Shewanella*, a non-fermenting bacterium, is generally considered a conditional pathogen capable of metabolizing various electron acceptors (Ding et al. 2021). While it exhibits resistance to stress, some strains may cause symptoms such as diarrhea and infections (Hau and Gralnick 2007). *Faecalibacterium*, a key microorganism in the intestines, is an important producer of butyrate in the colon (Ferreira-Halder et al. 2017). Its metabolites have anti-inflammatory effects, and they play a crucial role in regulating colonic immune health (Sokol et al. 2008). The salicylic acid produced by *Faecalibacterium* can reduce IL-8 levels by regulating NF-κB, significantly alleviating TNBS-induced colitis in mice (Miquel et al. 2015; Sokol et al. 2009). Our study also identified specific milk microbes in human milk, such as *Bifidobacterium* and *Staphylococcus*, varying among individuals (Lyons et al. 2022). The majority of specific milk microbes detected in our research are potentially beneficial microorganisms crucial for supporting intestinal health and immune development in newborn animals, ensuring their needs are met. Differences observed in specific milk microbes among sheep, goats, and humans may be attributed to distinct genetic backgrounds. While strict aseptic techniques were employed, further research is needed to explore similar patterns of specific milk microbes in different species or breeds, considering the potential influence of external contamination.

Our investigation delves into the intricate interplay between milk composition and the associated microbiota. Notably, in sheep milk, we observed a significant positive correlation between the relative abundance of *Lactobacillus* and the contents of total solids, SNF, protein, and casein. Intriguingly, *Bacteroide*s and *Bifidobacterium* exhibited a significant positive correlation with low lactose content, while *UCG-005*, *Bacteroides*, and *Bifidobacterium* demonstrated a significant positive correlation with galactose content. Additionally, 19 core genera, including *Prevotella*, displayed a positive correlation with lactose, galactose, and low lactose. These findings align with similar studies in sow, underscoring the consistency of the observed correlations (Chen et al. 2018c). Moreover, our research highlighted the fermentative capabilities of *Bifidobacterium* in milk, particularly in the fermentation of human milk oligosaccharides (HMOS) such as lactose and galactose, producing butyric acid and lactic acid and fostering its own growth (Katayama 2016). Certain HMOS were identified to possess antibacterial properties against *Streptococcus* (Ackerman et al. 2017). Further investigations indicated that the nutritional composition of milk may indeed exert a promotive and consistent influence on milk microbiota growth. For instance, anaerobic cocci, spore-forming bacteria, and acidophilic rods exhibited a positive correlation with protein content, whereas *Enterobacteriaceae* and *Actinobacteria* displayed a negative correlation with lactose content. *Staphylococcus* abundance in milk showed a negative correlation with fat content (Boix-Amorós et al. 2016). The study by Erica Kosmerl emphasized the interaction between the milk fat globule membrane (MFGM) and surface proteins of milk microbiota, regulating microbiota composition (Kosmerl et al. 2021). Monosaccharides in MFGM complexes were found to be utilized by lactic acid bacteria. Additionally, milk protein content, such as lactoferrin (LF), emerged as a crucial nutrient for the growth of beneficial bacteria like *Lactobacilli* and *Bifidobacteria* (Martinovic et al. 2013). Our research underscores the pivotal role of changes in milk nutrient composition in regulating milk microbiota composition.

The pre-weaning period for rumen microbiota colonization in ruminants is critical, with vertical transmission being a key factor influencing gastrointestinal microbiota development (Francino 2014). Milk microbiota, as a crucial link in vertical transmission, undergo dynamic changes during lactation stages (colostrum, transitional milk, and mature milk), holding biological significance for the establishment and maturation of gastrointestinal microbiota and the immune system in young animals (Cabrera-Rubio et al. 2012). Various factors, such as lactation stage, lifestyle, immunity, and diseases, contribute to changes in milk microbial composition, with lactation stage being the primary driver (Khodayar-Pardo et al. 2014). Our previous study revealed a gradual increase in rumen microbiota diversity on postnatal days (Lei et al. 2018). Significant differences in microbiota composition were observed between 10-day-old and 20-day-old goat kids, stabilizing after 28 days, indicating age-specific development (Li et al. 2019). The relative abundance *Bacteroidetes* and the prevalence of *Prevotella* and *Ruminococcaceae* increased with age (Zhang et al. 2019). Similar patterns were confirmed in comparable studies (Zhu et al. 2018). The relative abundance of *Alloprevotella* and *Moraxella* in Hu sheep’s rumen increased in the first 3 days post-birth and then decreased (Yin et al. 2021). Additionally, our study identified *Proteobacteria*, *Firmicutes*, *Actinobacteriota*, and *Bacteroidota* as the core phyla in sheep colostrum, transitional milk, and mature milk, akin to findings in human milk (Murphy et al. 2017), cow milk (Van Hese et al. 2022), and sows (Chen et al. 2018c). In sheep colostrum, *Proteobacteria* and *Firmicutes* displayed higher relative abundance than transitional and mature milk, transitioning to *Bacteroidota* with increased lactation days, mirroring patterns observed in lamb gastrointestinal microbiota colonization (Guo et al. 2020; Lei et al. 2018). Core genera in Pengbo sheep milk encompassed *Ralstonia*, *Lactobacillus*, *Streptomyces*, *Acinetobacter*, *Weissella*, and *Leuconostoc*, diverging from core genera in cow colostrum, such as *Acinetobacter*, *Pseudomonas*, *Enterobacteriaceae*, and *Lactococcus* (Boix-Amorós et al. 2016; Cabrera-Rubio et al. 2012; Chen et al. 2018b; Geng et al. 2021). In conclusion, our findings suggest both commonality and specificity in milk microbiota across species, with age playing a defining role.

Our investigation into the phenotypic functions of milk microbiota across different lactation stages in Tibetan sheep and goats reveals a nuanced interplay between microbial composition and functional roles. Utilizing BugBase for microbial phenotype prediction, we uncovered significant variations in microbial communities among the three defined milk types (Type 1, Type 2, and Type 3). Notably, anaerobic bacteria exhibited a marked prevalence in Type 2 milk, indicative of its unique adaptive landscape characterized by heightened metabolic versatility and possibly enhanced stress tolerance compared to Type 1 and Type 3 milks (Shi et al. 2024). Furthermore, our species-phenotype contribution analysis unveiled distinct microbial contributors to stress tolerance, potential pathogenicity, and biofilm formation across the milk types. In Type 1 milk, *Ralstonia* emerged as a dominant genus associated with stress tolerance and potential pathogenicity, underscoring its adaptive strategies in the colostrum-rich environment (Patel et al. 2017; Zhang et al. 2024). Notably, biofilm formation in Type 2 milk involved a diverse array including *Bifidobacterium* and *Ralstonia*. These insights highlight the dynamic and phenotype-specific interactions between milk microbiota and their functional roles throughout lactation. The observed variations underscore the adaptive strategies of milk-associated microbes, reflecting their evolutionary responses to the changing nutrient and immune environments within Tibetan sheep and goats’ milk. Our findings not only deepen the understanding of microbial ecology in mammalian milk but also emphasize the complex interplay between microbial composition and functional diversity in supporting early life nutrition and health. Future studies could explore how these microbial adaptations influence offspring development and health outcomes, further elucidating the intricate symbiosis between milk microbiota and mammalian hosts.

In conclusion, our long-term study on the dynamic changes in milk nutrient composition and microbial diversity in Tibetan goats and sheep revealed several key findings. The nutrient composition of milk from Tibetan goats and sheep exhibited a stage-wise decrease from lactation days 0 to 56, and complex correlations were observed among different nutrient components in the milk. Additionally, as lactation days increased, significant changes in the diversity and composition of milk microbiota were evident in Tibetan goats and sheep. These dynamic changes in milk nutrient composition and microbial diversity were found to be consistent, to some extent, with the developmental patterns of rumen microbiota in early lambs, indicating potential selection for immune-related microbial functions and reflecting changes in resource availability during the lactation period. Based on the age-specific composition of milk microbiota, we classified milk from lactation days 0 to 56 into three milk types: Type 1 (D0–D14), Type 2 (D14–D28), and Type 3 (D28–D56). Importantly, specific microbes were identified in the colostrum of goats and sheep, along with unique milk microbiota specific to each species. Overall, these findings provide valuable insights into the dynamic changes of milk nutrient composition and microbial diversity during early lactation in goats and sheep, and lay the foundation for the development and utilization of solid feed for lambs after weaning. The results have significant implications for improved management practices in the livestock industry, enhancing lamb health and productivity. Further research in this area may lead to targeted interventions and innovations in animal husbandry, contributing to more sustainable and efficient livestock production systems.

## Supplementary Information

Below is the link to the electronic supplementary material.

Supplementary file1 (PDF 496 KB)

Supplementary file2 (XLSX 1123 KB)

## Data Availability

The 16S rRNA sequencing data are available from the National Center for Biotechnology Information (NCBI) under accessions PRJNA1044939 respectively.
