# Supplementary material for: Age-specific composition of milk microbiota in Tibetan sheep and goats
Source: Appl Microbiol Biotechnol. 2024 Jul 9;108:411. doi: 10.1007/s00253-024-13252-w (PMC11233330; doi:10.1007/s00253-024-13252-w)
Supplement: Supplementary file 1 [file 253_2024_13252_MOESM1_ESM.pdf]

Supplementary Figures

Journal name: Applied Microbiology and Biotechnology

Manuscript Title: Age-Specific Composition of Milk Microbiota in Tibetan Sheep and Goats

The names of the authors: Xi Cao[1†]; Yumeng Fang[1†]; Pingcuo Bandan[2†]; Langda Suo[2]; Gesang Jiacuo[2]; Yujiang Wu[2]; Awang Cuoji[2]; Deqing Zhuoga[2]; Yulin Chen[1]; De Ji[2*]; Ciren Quzhen[2*]; Ke Zhang[1*]

[1] Key Laboratory of Animal Genetics, Breeding and Reproduction of Shaanxi Province, College of Animal Science and Technology, Northwest A&F University, Yangling, 712100, China;

[2] Institute of Animal Sciences, Tibet Academy of Agricultural and Animal Husbandry Sciences, Lhasa, 850009, China

[*]To whom correspondence should be addressed. Tel: +8618821639602; Email: kezhang@nwafu.edu.cn

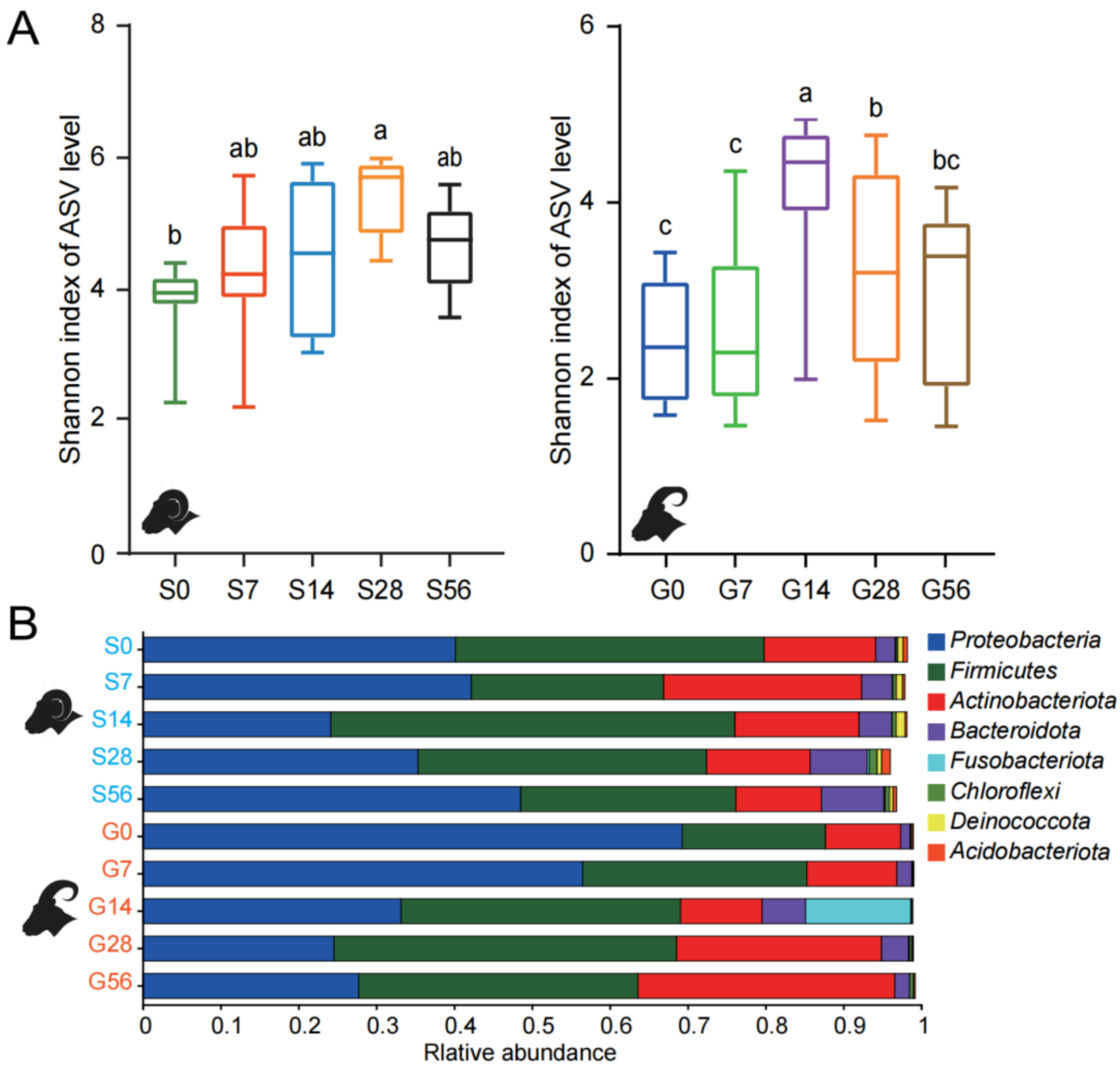

Supplementary Fig. 1. Analysis of Microbial Diversity and Composition in the Milk of Sheep and Goats during Lactation. (A) Detection of Shannon index for microbial communities in the milk of sheep and goats at the ASV Level. (B) Relative abundance changes of Phylum in the milk of sheep and goats. Same letters indicate non-significant differences, while different letters indicate significant differences.

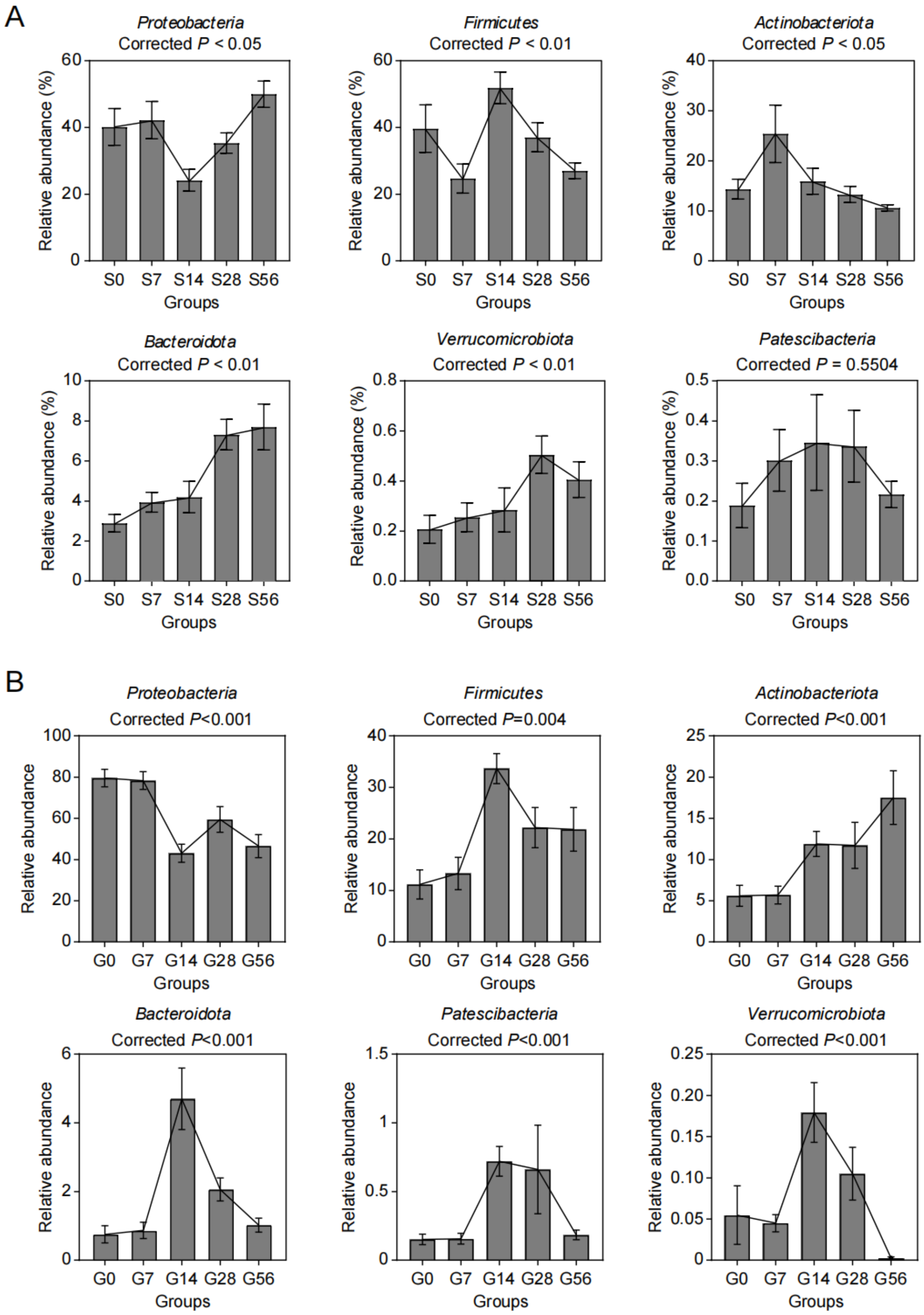

Supplementary Fig. 2. Relative abundance changes of shared phyla in the milk of sheep and goats. Comparison of the relative abundance changes of shared Phyla in the milk of sheep (A) and goats (B) with Wilcoxon Rank-Sum Test.

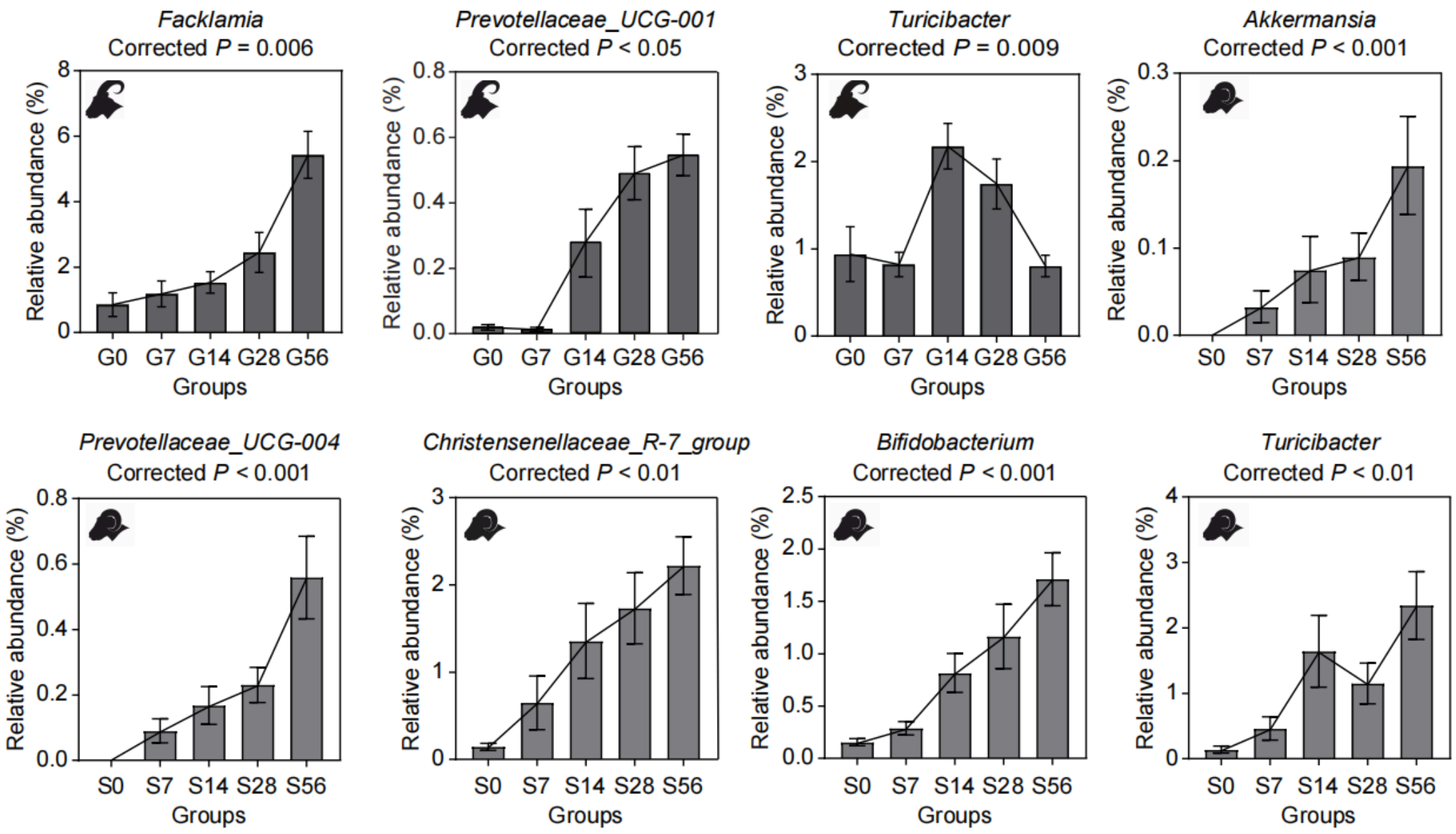

Supplementary Fig. 3. Milk microbiota in goats and sheep with genus-specific changes in relative abundance in milk. The relative abundance of some potential probiotic bacteria in the milk of goats and sheep increases from D0 to D56.
